# Changes in childhood stroke mortality from 1990 to 2019 in Brazil and its federative units

**DOI:** 10.1038/s41598-022-24761-x

**Published:** 2022-12-01

**Authors:** Laura Silveira Tanisaka, Fernando Rocha Oliveira, Luiz Vinicius de Alcantara Sousa, Luiz Carlos de Abreu, Fernando Adami, Laércio da Silva Paiva

**Affiliations:** 1 Faculdade de Saúde Pública da Universidade de São Paulo, São Paulo, SP Brazil; 2 School of Medicine, University of Limerick, Limerick, Ireland; 3 Present Address: Laboratório de Epidemiologia e Análise de Dados do Centro Universitário FMABC, Avenida Lauro Gomes, 2000 – Vila Sacadura Cabral, Santo André, SP 09060-870 Brazil

**Keywords:** Epidemiology, Stroke

## Abstract

This research analyzed the temporal trend of stroke mortality in children aged 0–14 years, from 1990 to 2019, in Brazil and its federative units. This ecological study used data from the Global Burden of Disease, a study led by the Institute for Health Metrics and Evaluation. Stroke definition considered the International Classification of Diseases according to codes G45, G46, and I60–I69. Age-standardized mortality rates and the mean annual percentage change (APC) in mortality rates were estimated. Stroke mortality trends decreased, with an APC of − 3.9% (95% CI − 4.5; − 3.3; p < 0.001). Reducing trends were found in all but two states, where they were stationary. Maranhão (− 6.5%; 95% CI − 7.6; − 5.4; p < 0.001) had the greatest reduction and Rondônia, the smallest (− 1.2%; 95% CI − 2.3; − 0.1, p = 0.027). Decrease was more important in children < 5 (− 5.8%; 95% CI − 6.3; − 5.2; p < 0.001) compared to 5–14 years old (− 2.1%; 95% CI − 2.9; − 1.3; p < 0.001); additionally, it was greater in girls (− 4.1%; 95% CI − 4.6; − 3.5; p < 0.001) than in boys (− 3.8%; 95% IC − 4.5; − 3.1; p < 0.001). Ischemic stroke had the highest APC (− 6.1%; 95% CI − 6.8; − 5.3; p < 0.001), followed by intracranial hemorrhage (− 5.3%; 95% CI − 6.1; − 4.5; p < 0.001) and subarachnoid hemorrhage (− 2.7%; 95% CI − 3.3; − 2.1; p < 0.001). Largest reductions were seen in states with more vulnerable socioeconomic contexts. The stationary trends and lowest APCs were concentrated in the northern region, which had greater impact of diseases and less favorable outcomes.

## Introduction

Perinatal and childhood stroke affects 3–25 patients per 100,000 each year, with the highest incidence relating to the neonatal period, with 1 in 4000 live births^1^. Globally, from 1990 to 2013, the number of prevalent pediatric cases has increased by approximately 35%^2^.

In childhood, stroke has a relatively rare association with risk factors that are common in adults, such as high systolic blood pressure, high body mass index and high fasting plasma glucose, as well as environmental pollution and smoking^3^. Instead, stroke in childhood is mainly related to coagulation disorders, infections, cardiac defects, vascular anomalies, sickle cell anemia, and metabolic or genetic diseases^1^. In children, delays in the suspicion and recognition of the diagnosis are common^4^; and when compared to adults, clinical presentation is nonspecific and may include hemiparesis, weakness, altered mental status, headache, and seizures^5^. Consequently, those differences must be considered when developing new strategies for prevention and treatment, since they represent remarkable clues to identify childhood stroke^3,6^. In addition, delayed treatment has a substantial economic impact on childhood stroke. As an outcome in this age group, stroke survivors commonly present with cognitive, skill, processing or memory deficiencies that can influence their quality of personal and family life^7,8^. One study showed that the direct medical costs during the 5 years post-stroke are 15 times higher in children with stroke than in age-matched children without stroke^9^. In addition, stroke-associated neurological deficits may persist into adulthood. Thus, indirect costs linked to productivity loss from neurological sequelae should also be considered^8,10^.

Overall, childhood stroke mortality has decreased^2,11^. The Global Burden of Disease study has reported a significant decline in the childhood stroke death rates worldwide, between 1990 and 2013, with an important increase in the absolute number of prevalent cases^2^. Moreover, a previous study from 1979 to 1998 found that childhood mortality from stroke declined by 58% overall^11^. Although older studies suggest 20% mortality after stroke in children^12^, recent literature showed lower rates. A population-based retrospective cohort from the United States found an overall mortality at the time of hospital discharge of 4% while the Canadian Pediatric Stroke Registry revealed a stroke-specific mortality of 5% over a median follow-up 3 years in 2012 and 2017, respectively^13,14^.

Recently, worldwide development of new medical centers and professional teams focused on stroke treatment in children has helped reduce the associated mortality and morbidity rates^5,6,11^. Simultaneously, access to appropriate imaging modalities allows adequate celerity in stroke care for neonates and children^4,15^. In Brazil, the prevalence and frequency of hospitalizations for pediatric stroke are progressively increasing^16^. The increasing number of cases may be linked to several factors^2^. However, there are a lack of studies on the temporal pattern of childhood stroke mortality in Brazil, as well as its association with risk factors, especially socioeconomic factors, according to federative units.

Thus, considering the increasing prevalence of pediatric stroke, both in Brazil and worldwide, as well as the lack of literature showing temporal mortality behavior from childhood stroke in Brazil, this study is critical. In 2013, the Brazilian Ministry of Health reported the lack of childhood stroke statistics in Brazil^17^. Above all, estimates of stroke influence are necessary for planning future research and evidence-based health care strategies aimed at the prevention and treatment of stroke, especially in children. Research can lead to significant changes in public healthcare policy, such as the implementation of mechanical thrombectomy in the national public healthcare system for patients > 18 years old, supported by the RESILIENT trial^18^. Due to the challenging nature of diagnosis and management, our objective was to analyze changes in temporal trends of stroke mortality in children aged 0–14 years from 1990 to 2019, in Brazil and its respective federative units.

## Methods

This ecological study in Brazil evaluated the temporal trend of stroke mortality in patients aged 0–14 years, who were diagnosed with stroke between 1990 and 2019, by means of a secondary analysis of data. Data on stroke mortality were extracted from the Global Burden of Disease (GBD) system^19^, which is considered the most comprehensive worldwide observational epidemiological study so far. The GBD is led by the Institute for Health Metrics and Evaluation (IHME) and by tracking progress within and between countries, GBD provides an important and open access informative tool. Data capture premature death and disability from more than 350 diseases and injuries in 195 countries, by age and sex, allowing comparisons over time, across age groups, and among populations. It can be accessed online (http://www.healthdata.org/)—and all input data is identified via the Global Health Data Exchange website^20^. The study is performed in compliance with Guidelines for Accurate and Transparent Health Estimates Reporting (GATHER) guidelines for reporting health estimates^19^. Authors had full access to all the data in the study and take responsibility for its integrity and the data analysis.

The presented research considered the International Classification of Diseases (ICD-10), including patients diagnosed with stroke not specified as ischemic or hemorrhagic, by the ICD-10 codes G45, G46, and I60–I69, from 1990 to 2019. Data collection included the following: sex, age group (< 5 and 5–14 years old), stroke subtype (ischemic, subarachnoid hemorrhagic, and intracerebral hemorrhagic), and Brazilian federative unit (Acre, Amapá, Amazonas, Bahia, Ceará, Espírito Santo, Goiás, Maranhão, Mato Grosso, Mato Grosso do Sul, Minas Gerais, Pará, Paraíba, Paraná, Pernambuco, Piauí, Rio de Janeiro, Rio Grande do Norte, Rio Grande do Sul, Rondônia, Roraima, Santa Catarina, São Paulo, Sergipe, Tocantins, Federal District).

To analyze stroke by age, we divided stroke sample into two age at stroke groups. Once the GBD databases group together individuals aged 15 to 19 years old and childhood stroke usually includes up to the age of 18, we focused our search in those aged 0–14 years old. Moreover, in the literature, there are some inconsistencies regarding the cut-off age to define stroke in “young adults”, with some studies using the range of 15–49 years^21,22^.

It is also important to note that the GBD databases does not provide aggregated rates, that would be necessary when analyzing regions rather than states. The analysis by federative units provides more detailed data, since it considers a geographic area that is even more delimited compared to regional division.

### Statistical analysis

To describe stroke mortality trends, time series construction rates were calculated using the Prais-Winsten linear regression model, proposed by Antunes et al.^23^ which allowed first-order autocorrelation corrections to be performed on values organized by time. This is a great and trustable regression model to study temporal trends. We estimated the slope (β), its respective probability (*p*), and predictive capacity of the model (r^2^). Temporal trends were estimated according to the patterns of location, sex, age, and year, with a confidence interval of 95%. The statistical program used was Stata, version 14.0.

Indicator trends can be considered increasing, decreasing, or stationary. The β value of the regression is positive when there is an increasing trend, negative when there is a decreasing trend, and null when there is a stationary trend; that is, there is no significant difference between its value and zero^23^.

### Ethics declarations

The data used in this research come from a public database of international scope, which is unrestricted and allows public access. This means that there is no need for evaluation by the Research Ethics Committee, according to the Brazilian National Health Council Resolution no. 510/2016.

## Results

In Brazil, from 1990 to 2019, temporal trends in mortality rates from unspecified stroke in children ≤ 14 years showed a reduction pattern with an annual percentage change (APC) of − 3.9% (p < 0.001) (Table 1). Data analysis demonstrated a statistically significant decrease for both sexes. There was a greater reduction of mortality in girls (− 4.1%; 95% CI − 4.6; − 3.5) than in boys (− 3.8%; 95% CI − 4.5; − 3.1) (Table 1).

**Table 1:** Mortality rate temporal variation according to sex, age group and locations from 1990–2019.

Characteristics																															APC (CI 95%)	r^2^	p	Trend
Sex	1990	1991	1992	1993	1994	1995	1996	1997	1998	1999	2000	2001	2002	2003	2004	2005	2006	2007	2008	2009	2010	2011	2012	2013	2014	2015	2016	2017	2018	2019	1990–2019			
Male	1.34	1.27	1.20	1.12	1.08	1.05	1.02	0.98	0.93	0.92	0.89	0.90	0.87	0.84	0.83	0.82	0.82	0.81	0.79	0.77	0.73	0.69	0.67	0.63	0.58	0.53	0.50	0.48	0.45	0.43	− 3.8 (− 4.5; − 3.1)	0.58	< 0.001	D
Female	1.19	1.15	1.08	1.05	1.01	0.95	0.91	0.87	0.83	0.80	0.78	0.77	0.75	0.72	0.70	0.69	0.68	0.67	0.66	0.64	0.60	0.58	0.54	0.50	0.46	0.43	0.41	0.39	0.37	0.35	− 4.1 (− 4.6; − 3.5)	0.65	< 0.001	D
**Age group (years)**																																		
< 5	2.46	2.36	2.25	2.11	1.98	1.88	1.78	1.67	1.57	1.50	1.44	1.41	1.34	1.29	1.23	1.19	1.14	1.08	1.02	0.95	0.88	0.82	0.75	0.69	0.63	0.57	0.54	0.51	0.47	0.44	− 5.8 (− 6.3; − 5.2)	0.87	< 0.001	D
5–14	0.70	0.67	0.63	0.61	0.62	0.60	0.60	0.58	0.57	0.57	0.55	0.56	0.56	0.55	0.54	0.55	0.57	0.58	0.58	0.58	0.56	0.54	0.53	0.50	0.47	0.43	0.41	0.40	0.38	0.37	− 2.1 (− 2.9; − 1.3)	0.22	< 0.001	D
**Stroke subtypes**																																		
Subarachnoid Hemorrhage (SAH)	0.57	0.55	0.53	0.51	0.52	0.49	0.50	0.47	0.48	0.46	0.45	0.44	0.45	0.43	0.42	0.43	0.42	0.42	0.42	0.41	0.39	0.36	0.38	0.32	0.34	0.29	0.30	0.26	0.28	0.26	− 2.7 (− 3.3; − 2.1)	0.45	< 0.001	D
Intracerebral Hemorrhage (IH)	0.54	0.57	0.50	0.46	0.44	0.42	0.37	0.40	0.35	0.34	0.33	0.33	0.30	0.32	0.29	0.29	0.28	0.28	0.25	0.26	0.22	0.23	0.19	0.15	0.17	0.21	0.13	0.14	0.12	0.12	− 5.3 (− 6.1; − 4.5)	0.45	< 0.001	D
Ischemic Stroke (IS)	0.13	0.12	0.10	0.11	0.09	0.09	0.07	0.08	0.06	0.06	0.05	0.06	0.05	0.05	0.05	0.05	0.05	0.05	0.04	0.04	0.04	0.04	0.03	0.04	0.03	0.03	0.02	0.03	0.02	0.02	− 6.1 (− 6.8; − 5.3)	0.86	< 0.001	D
**Brazil and federative units**																																		
Brazil	1.27	1.21	1.14	1.08	1.05	1.00	0.97	0.93	0.88	0.86	0.84	0.83	0.81	0.79	0.76	0.76	0.76	0.74	0.72	0.70	0.67	0.63	0.60	0.56	0.52	0.48	0.46	0.43	0.41	0.39	− 3.9 (− 4.5; − 3.3)	0.6	< 0.001	D
Acre	1.22	1.17	1.15	1.11	0.98	0.92	1.06	1.05	1.01	0.94	0.89	0.97	0.90	0.95	0.84	0.76	0.65	0.68	0.67	0.67	0.68	0.65	0.61	0.58	0.55	0.51	0.49	0.46	0.44	0.43	− 3.5 (− 4.0; − 2.9)	0.84	< 0.001	D
Alagoas	3.57	3.37	3.06	2.73	2.49	2.25	2.06	1.88	1.68	1.62	1.58	1.63	1.55	1.48	1.43	1.41	1.40	1.39	1.35	1.31	1.23	1.14	1.03	0.92	0.80	0.70	0.65	0.59	0.55	0.52	− 6.4 (− 7.5; − 5.2)	0.8	< 0.001	D
Amapá	0.51	0.54	0.54	0.59	0.56	0.56	0.53	0.47	0.44	0.43	0.41	0.42	0.38	0.36	0.31	0.28	0.28	0.32	0.40	0.42	0.43	0.40	0.41	0.43	0.46	0.46	0.47	0.46	0.42	0.40	− 0.8 (− 2.5; 0.9)	0.19	0.333	S
Amazonas	0.70	0.63	0.62	0.60	0.60	0.58	0.56	0.56	0.56	0.55	0.57	0.59	0.49	0.46	0.44	0.39	0.42	0.44	0.48	0.52	0.53	0.53	0.51	0.50	0.48	0.46	0.47	0.47	0.42	0.40	− 1.6 (− 2.6; − 0.7)	0.06	0.002	D
Bahia	1.55	1.51	1.41	1.29	1.21	1.20	1.19	1.19	1.16	1.18	1.15	1.19	1.17	1.13	1.12	1.14	1.14	1.12	1.07	1.02	0.96	0.91	0.85	0.77	0.69	0.63	0.59	0.55	0.53	0.51	− 3.8 (− 4.9; − 2.6)	0.41	< 0.001	D
Ceará	2.75	2.50	2.20	1.92	1.73	1.62	1.52	1.41	1.37	1.32	1.28	1.29	1.27	1.24	1.15	1.15	1.15	1.16	1.16	1.06	1.00	0.93	0.84	0.78	0.68	0.60	0.54	0.49	0.47	0.45	− 6.0 (− 7.2; − 4.8)	0.7	< 0.001	D
Distrito Federal	0.49	0.48	0.47	0.49	0.48	0.45	0.42	0.37	0.33	0.31	0.30	0.31	0.30	0.28	0.27	0.28	0.28	0.27	0.27	0.28	0.27	0.26	0.23	0.22	0.21	0.21	0.20	0.20	0.19	0.18	− 3.3 (− 4.0; − 2.7)	0.65	< 0.001	D
Espírito Santo	0.87	0.80	0.79	0.78	0.76	0.69	0.63	0.60	0.60	0.59	0.60	0.63	0.62	0.63	0.63	0.66	0.71	0.72	0.65	0.63	0.62	0.59	0.57	0.55	0.51	0.46	0.43	0.40	0.40	0.38	− 2.7 (− 3.8; − 1.5)	0.11	< 0.001	D
Goiás	0.70	0.64	0.59	0.54	0.53	0.50	0.45	0.43	0.39	0.38	0.35	0.34	0.34	0.32	0.32	0.31	0.30	0.29	0.29	0.31	0.33	0.34	0.33	0.31	0.32	0.31	0.29	0.27	0.26	0.25	− 3.4 (− 4.7; − 2.1)	–	< 0.001	D
Maranhão	4.81	4.68	4.48	4.21	3.83	3.58	3.40	3.19	3.01	2.86	2.76	2.83	2.79	2.66	2.58	2.45	2.30	2.14	1.99	1.83	1.63	1.43	1.29	1.15	0.99	0.89	0.82	0.77	0.72	0.69	− 6.5 (− 7.6; − 5.4)	0.82	< 0.001	D
Mato Grosso	0.67	0.68	0.65	0.74	0.78	0.77	0.71	0.69	0.63	0.58	0.53	0.53	0.53	0.53	0.52	0.51	0.49	0.48	0.46	0.46	0.42	0.42	0.44	0.42	0.38	0.35	0.34	0.32	0.31	0.30	− 2.9 (− 3.6; − 2.2)	0.64	< 0.001	D
Mato Grosso do Sul	0.62	0.63	0.60	0.60	0.59	0.57	0.57	0.53	0.49	0.48	0.47	0.48	0.47	0.48	0.48	0.47	0.45	0.43	0.45	0.45	0.40	0.38	0.39	0.37	0.34	0.31	0.31	0.27	0.27	0.25	− 3.0 (− 3.7; − 2.2)	0.52	< 0.001	D
Minas Gerais	0.71	0.72	0.71	0.70	0.69	0.68	0.66	0.63	0.63	0.65	0.61	0.61	0.59	0.59	0.58	0.57	0.56	0.55	0.52	0.51	0.49	0.47	0.44	0.40	0.38	0.35	0.34	0.33	0.30	0.29	− 3.1 (− 4.0; − 2.2)	0.22	< 0.001	D
Pará	0.87	0.93	0.92	0.89	0.88	0.85	0.90	0.95	0.97	0.95	0.93	1.01	1.05	1.02	0.98	0.97	0.98	0.98	0.97	0.92	0.88	0.83	0.79	0.75	0.72	0.67	0.63	0.58	0.55	0.52	− 1.8 (− 3.2; − 0.3)	0.07	0.019	D
Paraíba	1.78	1.74	1.66	1.56	1.46	1.38	1.27	1.17	1.09	1.01	0.94	0.93	0.91	0.86	0.81	0.78	0.78	0.77	0.74	0.70	0.65	0.65	0.63	0.56	0.49	0.44	0.48	0.49	0.40	0.38	− 5.0 (− 5.4; − 4.5)	0.09	< 0.001	D
Paraná	0.57	0.54	0.52	0.53	0.55	0.51	0.49	0.47	0.47	0.46	0.45	0.46	0.48	0.48	0.47	0.49	0.51	0.51	0.50	0.49	0.45	0.44	0.43	0.41	0.38	0.35	0.34	0.33	0.31	0.30	− 2.1 (− 3.1; − 1.1)	0.35	< 0.001	D
Pernambuco	1.66	1.53	1.36	1.17	1.22	1.23	1.25	1.23	1.19	1.18	1.17	1.21	1.17	1.09	1.09	1.08	1.10	1.09	1.06	0.99	0.92	0.89	0.82	0.70	0.62	0.58	0.53	0.49	0.47	0.44	− 4.4 (− 5.8; − 2.9)	0.42	< 0.001	D
Piauí	2.29	2.11	1.94	1.74	1.54	1.44	1.39	1.32	1.27	1.25	1.23	1.29	1.29	1.25	1.28	1.24	1.22	1.19	1.13	1.06	0.99	0.94	0.87	0.82	0.81	0.76	0.70	0.64	0.60	0.58	− 4.6 (− 5.6; − 3.5)	0.69	< 0.001	D
Rio de Janeiro	1.08	1.01	1.00	1.02	1.03	0.92	0.88	0.80	0.75	0.72	0.66	0.59	0.56	0.56	0.56	0.56	0.56	0.57	0.60	0.60	0.58	0.58	0.57	0.55	0.51	0.46	0.47	0.45	0.42	0.40	− 3.3 (− 4.3; − 2.3)	0.28	< 0.001	D
Rio Grande do Norte	1.34	1.31	1.23	1.12	1.09	1.08	1.04	0.97	0.90	0.87	0.83	0.84	0.78	0.75	0.71	0.71	0.70	0.67	0.65	0.61	0.59	0.58	0.56	0.52	0.49	0.45	0.41	0.38	0.36	0.34	− 4.6 (− 5.1; − 4.0)	0.82	< 0.001	D
Rio Grande do Sul	0.59	0.59	0.55	0.55	0.57	0.57	0.53	0.48	0.47	0.45	0.45	0.45	0.42	0.41	0.40	0.38	0.37	0.37	0.37	0.37	0.37	0.37	0.38	0.36	0.37	0.35	0.33	0.33	0.31	0.30	− 2.3 (− 2.7; − 1.8)	0.7	< 0.001	D
Rondônia	0.54	0.51	0.48	0.48	0.46	0.44	0.46	0.53	0.54	0.60	0.57	0.54	0.56	0.50	0.43	0.40	0.39	0.40	0.46	0.51	0.52	0.46	0.45	0.45	0.46	0.45	0.41	0.39	0.37	0.35	− 1.2 (− 2.3; − 0.1)	0.29	0.027	D
Roraima	0.36	0.32	0.33	0.37	0.36	0.28	0.30	0.37	0.38	0.37	0.34	0.38	0.36	0.37	0.34	0.33	0.35	0.37	0.36	0.33	0.29	0.30	0.34	0.35	0.33	0.34	0.34	0.31	0.30	0.29	− 0.4 (− 0.9; 0.2)	0.35	0.201	S
São Paulo	0.69	0.64	0.63	0.66	0.68	0.69	0.68	0.65	0.59	0.58	0.57	0.50	0.46	0.44	0.42	0.43	0.45	0.44	0.43	0.44	0.44	0.42	0.42	0.42	0.41	0.37	0.36	0.35	0.34	0.33	− 2.6 (− 3.2; − 1.9)	0.5	< 0.001	D
Santa Catarina	0.61	0.60	0.59	0.57	0.56	0.54	0.53	0.49	0.44	0.44	0.45	0.45	0.45	0.46	0.44	0.44	0.45	0.44	0.44	0.42	0.38	0.38	0.40	0.36	0.31	0.29	0.30	0.29	0.27	0.26	− 2.8 (− 3.5; − 2.0)	0.55	< 0.001	D
Sergipe	1.70	1.58	1.39	1.29	1.25	1.18	1.09	1.03	0.98	0.92	0.90	0.87	0.83	0.80	0.76	0.71	0.67	0.70	0.74	0.72	0.71	0.67	0.62	0.55	0.48	0.45	0.44	0.43	0.39	0.38	− 4.9 (− 5.7; − 4.1)	0.75	< 0.001	D
Tocantins	1.61	1.47	1.43	1.36	1.27	1.22	1.21	1.21	1.12	1.08	1.07	1.12	1.08	1.02	0.97	0.93	0.91	0.92	0.90	0.88	0.80	0.72	0.69	0.67	0.58	0.51	0.48	0.45	0.42	0.40	− 4.6 (− 5.7; − 3.6)	0.59	< 0.001	D

*APC* annual percentage change, *r*^*2*^ predictive capacity of the model, *p* probability, *D* decreasing trend, *S* stationary trend.

When investigating by age group, a decrease in temporal trend was observed in both groups, although the reduction was larger in children < 5 years old, with a rate of − 5.8% per year (− 5.8; 95% CI − 6.3; − 5.2), in relation to children aged 5–14 years, with a reduction of − 2.1% per year (− 2.1; 95% CI − 2.9; − 1.3) (Table 1).

**Figure 1:**
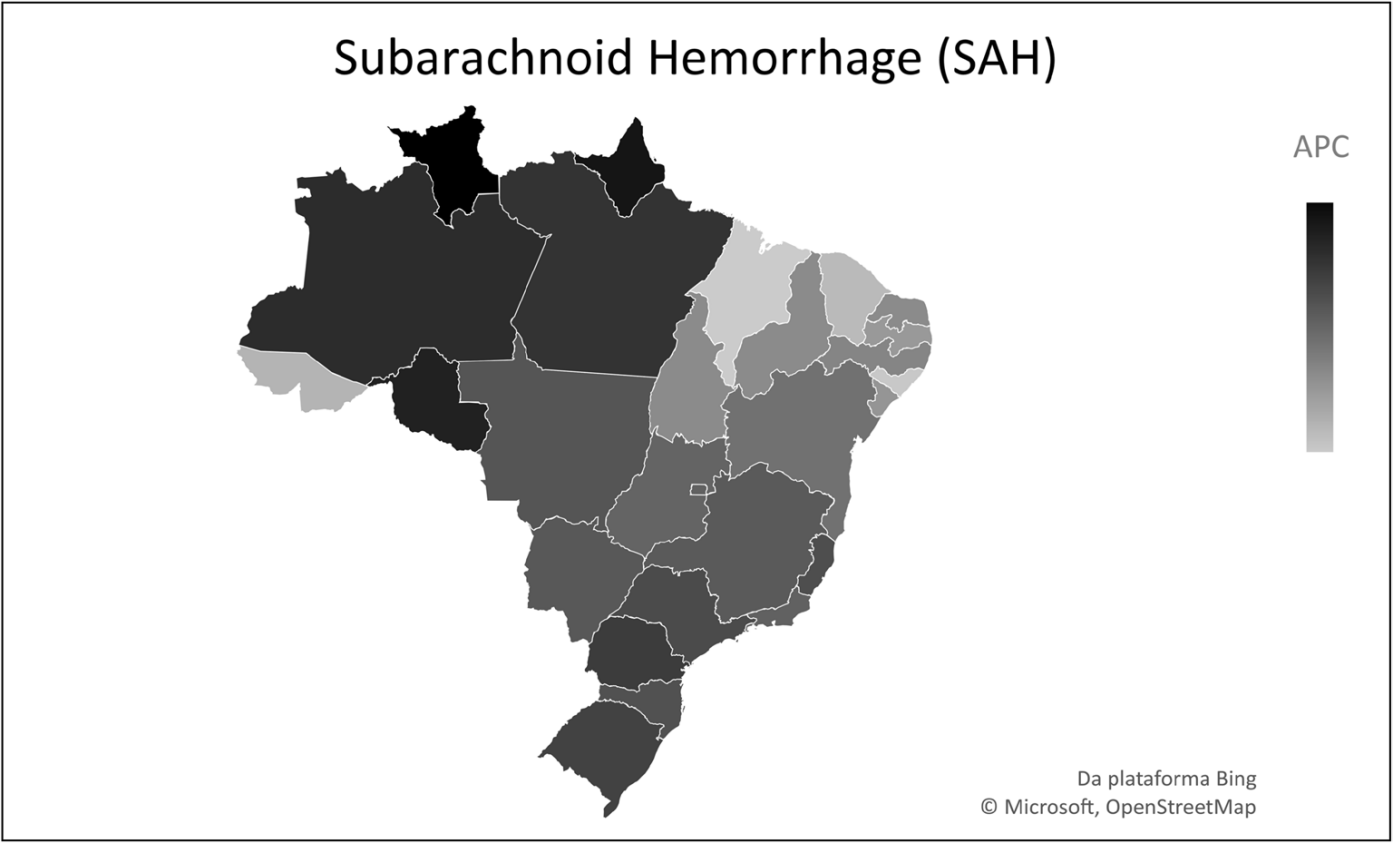
Temporal trend of mortality from childhood subarachnoid hemorrhage (SAH), from 1990–2019, in Brazilian Federative Units. *APC* annual percentage change.

**Figure 2:**
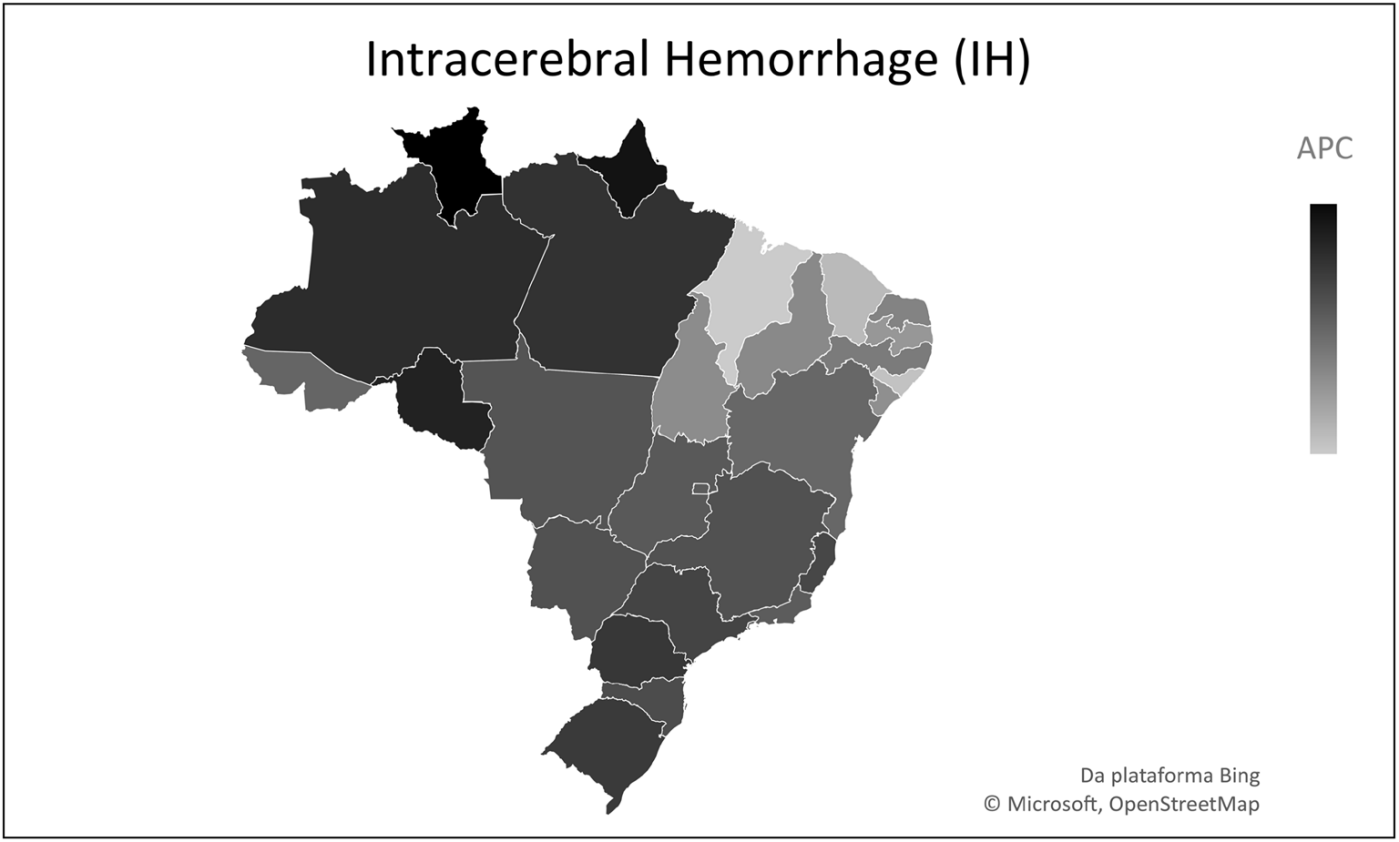
Temporal trend of mortality from childhood intracerebral hemorrhage (IH), from 1990–2019, in Brazilian Federative Units. *APC* annual percentage change.

**Figure 3:**
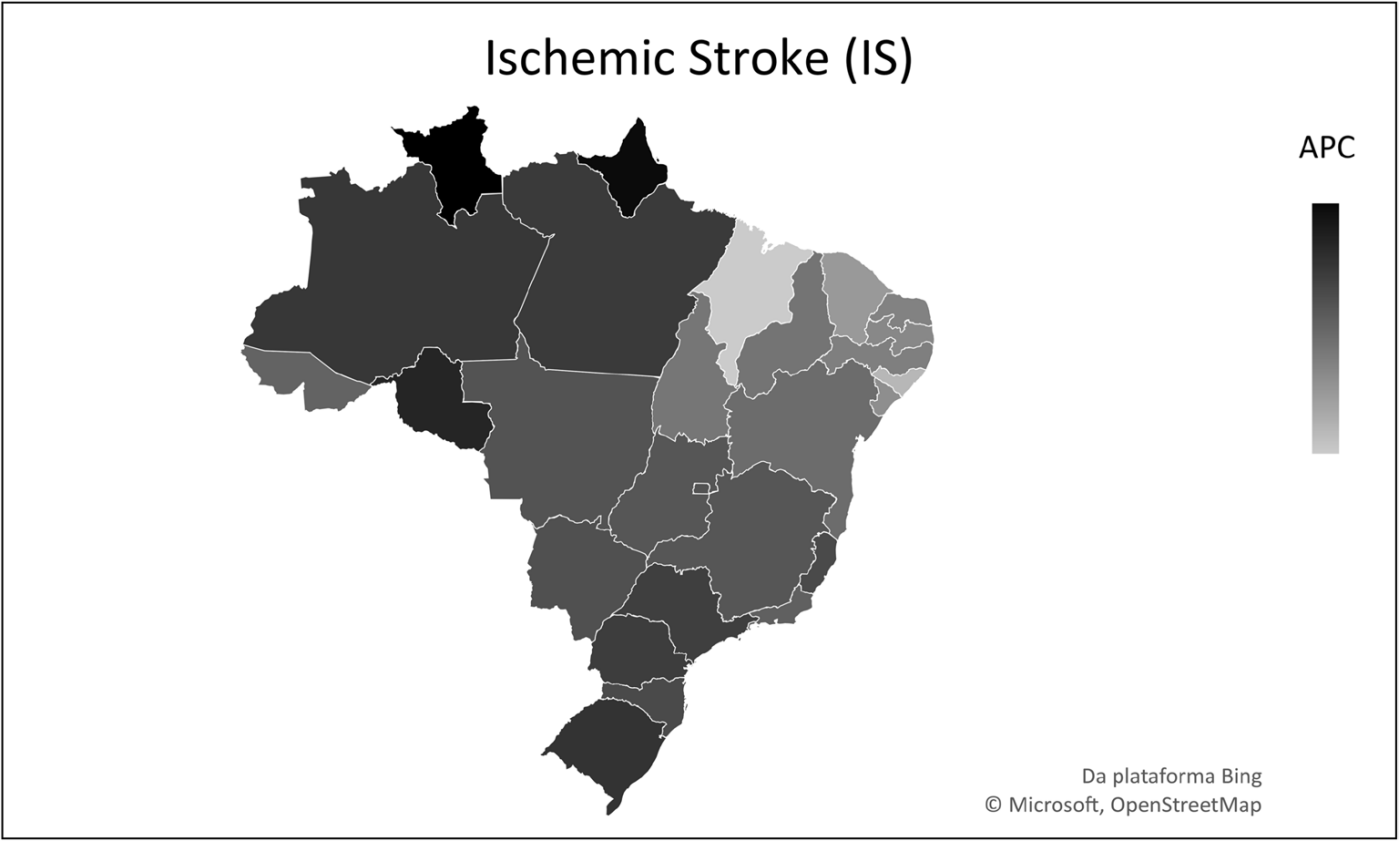
Temporal trend of mortality from childhood ischemic stroke (IS), from 1990–2019, in Brazilian Federative Units. *APC* annual percentage change.

The analysis according to location showed that, of the 27 Brazilian federative units, only two showed stationary trends (p > 0.05): Amapá and Roraima, whereas the other 25 presented with a reduction pattern (Table 1). Of these, Maranhão had the highest APC (− 6.5%), followed by Alagoas (− 6.4%). At the same time, the federative units with the lowest mortality reduction were Rondônia (− 1.2%) and Amazonas (− 1.6%) (Table 1) (Figs. 1, 2, 3).

In addition, the evaluation of mortality behavior by stroke subtype showed a decreasing trend during the study period. In Brazil, ischemic stroke (IS) was the subtype with the highest APC (− 6.1%), followed by intracranial hemorrhage (IH) (− 5.3%), and subarachnoid hemorrhage (SAH) (− 2.7%). As for the federative units, Amapá was the only unit to present a stationary trend (*p* > 0.05) for all subtypes. Among the other subtypes, in which there was a decrease in mortality for IS, the highest APC was in Maranhão (− 9.2%), followed by Alagoas (− 8.5%) and Ceará (− 7.5%) (see Supplementary Table S1 online). The same pattern was observed for IH, with the greatest reduction in Maranhão (− 8.3%), followed by Alagoas (− 8.0%) and Ceará (− 7.7%) (see Supplementary Table S2 online). Similarly, for SAH, the largest drop was associated with Maranhão (− 6.5%), followed by Alagoas (− 6.4%), and Ceará (− 6.0%) (see Supplementary Table S3 online). In contrast, smallest reductions were related to Roraima and Rondônia.

## Discussion

The main findings of the present study include the following: (1) in Brazil, from 1990–2019, temporal trends in mortality rates from unspecified stroke in children ≤ 14 years showed a reduction pattern; (2) of the 27 Brazilian federative units, only two showed stationary trends: Amapá and Roraima, and decrease was not homogeneous among the states; (3) smallest reductions were related to Roraima and Rondônia when evaluated by stroke subtypes; (4) there was a greater reduction of mortality in girls than in boys and in children < 5 years old in relation to children aged 5–14 years.

The evaluation of the temporal behavior of mortality from pediatric stroke in Brazil allows us to highlight a decreasing mortality trend between 1990 and 2019. Our data are consistent with the findings of other studies, which indicate a reduction in stroke mortality rate in children^2,11^.

There are factors possibly associated with this reduction, such as the improvement of neuroimaging diagnostic techniques, as well as increasing agility in access, due to their crucial nature in the efficient care of acute stroke in children^1,15^. In Brazil, stroke care for children follows a hierarchical and organized system. Health prevention occurs in primary health care units and through telemedicine; prehospital care is related to emergency mobile component; hospital care is promoted to carry out the treatment; and the after acute hospital care includes home care, rehabilitation and follow-up^24^. Moreover, it is important to note that, as a survey based on secondary data, the possibility of underreported deaths from this condition should be considered.

Furthermore, in the Brazilian context, it is interesting to observe the association between the reduction in morbidity and mortality from cardiovascular diseases and stroke, and the availability of a public health system that includes primary and secondary preventive care^25^. The expansion of the Family Health Strategy (FHS), the main primary health care programme of the country, relates to lower mortality rates in children, especially to post-neonatal and infant mortality^26^. Still, primary prevention of childhood stroke has not been achieved, except in high-risk situations such as sickle cell disease in which long-term blood transfusion therapy may be indicated, for example. In general, the treatment is focused on the prevention of recurrent stroke, and strongly depends on the underlying etiology^1,27^. However, the expansion of the Family Health Strategy relates to lower mortality rates in children, especially to post-neonatal and infant mortality^26^. In this regard, establishing health behaviors during the follow-up may be a relevant strategy to reduce the risk of obesity and sedentary lifestyle in order to protect children from recurrent strokes later in life^1^.

In our findings related to sex, we noticed a similarity with other studies in the literature. Our study showed a greater reduction of mortality in girls (− 4.1%) than in boys (− 3.8%). Krishnamurthi et al. in turn, using available global data on stroke incidence, prevalence, and mortality to assess differences and changes between developed and developing countries during the period from 1990 to 2013, found a substantial reduction in mortality rate, and that globally, boys had higher rates of stroke than girls, in 2013^2^.

Still, data analysis showed that, in relation to age groups, and considering stroke and its subtypes, the temporal trend was mostly decreasing in children < 5 years of age. This pattern is possibly associated with a higher incidence of the disease during the perinatal period, which occurs between 28 weeks of gestational age and 28 days of postnatal life^1^, and can intensify the attention required from health professionals during care. Detection can also be aided by the increased perinatal risk of stroke because, according to the literature, although this clinical condition involves multifactorial mechanisms, there is a strong correlation between stroke and congenital heart disease, prematurity, coagulation, trauma, asphyxia, and other neonatal pathologies^1,7,8^. In addition, maternal conditions such as a history of infertility, thrombophilia, preeclampsia, infections, and complications during pregnancy or childbirth are risk factors^7,8^.

Moreover, some studies have stated that the risk of stroke peaks during childhood, being higher in children < 1 year, followed by a considerable decline, which increases again during late adolescence. Simultaneously, the clinical presentation of acute IS may involve seizures more frequently than other subtypes. Thus, identification of the condition and early introduction of support measures, such as oxygen, fluids, and the correction of anemia, could be contributing to the reduction in mortality, especially related to acute IS. The literature also indicates that the same pattern of age distribution is observed in SAH, with an increased incidence in adolescents^1,8^.

In 1970, a new division into Macro regions was created in Brazil, and resulted in the following denominations: North Region, Northeast Region, Southeast Region, South Region and Midwest Region, which are considered until the present moment^28^. A national study conducted between 2002 and 2009 found that income inequality trends were independently associated with stroke mortality rate trends, even after adjusting for economic growth and other covariates^29^. In our study, stationary trends and the smallest annual reductions in childhood stroke mortality were related to states in the North Region: Amapá, Rondônia, Roraima, and Amazonas. This finding accords with other evidence in the literature, since the north portion of the country, especially in relation to the south and southeast, is a region with greater disease impacts with more unfavorable outcomes^30^.

Although our results indicate that most of the Brazilian federative units showed a decreasing mortality pattern from a stroke in children, the decrease varied heterogeneously among states. From this perspective, Maranhão and Alagoas had the highest annual reduction rates for all stroke subtypes in relation to other federative units. It is known that between 1990 and 2016, Alagoas had an expressive increase in life expectancy, 9.5 years, while Maranhão rose only 5.3 years. A national review that analyzed different geographical areas of Brazil showed that studies have found higher rates, or higher percentages of increased mortality rates, including stroke, in areas considered to be more impoverished, vulnerable, or have less socioeconomic development^31^. Other study on leading causes of death in Brazil observed that between 2000 and 2012 the mortality rates due to cerebrovascular diseases, hypertensive diseases and circulatory system diseases were influenced by socioeconomic factors. There was a significant inverse association between socioeconomic factors and mortality rates^32^. In view of this, it is possible that improvement in socioeconomic conditions of these localities, as evidenced by increasing sociodemographic indices^33^ over the period, influenced the high percentage reduction in childhood stroke mortality; however, at the same time, inefficient or inadequate data recording may have favored underreported deaths.

In relation to socioeconomic context, access to information is an important factor, in children and adults, in which less understanding of stroke is associated with lower socioeconomic and educational attainment levels^33^. Childrens’ knowledge of stroke risk factors and symptoms was lower in the socioeconomically vulnerable and in those with poor academic performance^34,35^. This impairs the seeking of medical services, in the face of an acute clinical condition, in which early diagnosis and treatment are imperative. This failure to recognize a stroke is especially relevant given the frequency at which stroke, among other neurological emergencies, can appear. Studies on pediatric stroke care protocols have already shown that of all pediatric neurological emergencies, stroke represents up to 45%, emphasizing the need for immediate assessment and support to optimize care and prevent recurrence^5,36^.

Socioeconomic conditions also influence prognosis after stroke. In a Danish data study with a national registry from 2003 to 2012 (n = 60,503 strokes), long-term, but not short-term, mortality after stroke was inversely related to income for all causes of death. There was a 5.7% absolute difference in mortality between the lowest and highest income groups five years after stroke. It is possible that social inequality is experienced, not in the ability to survive stroke, but in developing new diseases that subsequently lead to death^37^. Furthermore, in a large, multinational, prospective cohort of children with IS, low income was associated with worse neurologic outcomes compared to higher income levels. The study hypothesized that in lower-income settings, more severe/noticeable stroke symptoms might be necessary to justify seeking medical help, although root causes were not clear^38^.

Our findings contribute to a better understanding of the temporal trends in childhood stroke mortality. However, studies based on official mortality data should consider the inherent limitations of mortality surveillance systems and official health statistics. Similarly, incorrect registration of ICD codes to classify stroke can underestimate the number of found cases^39^. Other limitations that is worthy the note is that we analyzed stroke mortality based on rates; and the GBD databases does not provide data on risk factors or socioeconomic aspects related to stroke in children. Also, our analysis based on age groups did not include the > 14 and < 18 years old interval, which may have resulted in underreported cases of childhood stroke.

Furthermore, given that the incidence of hospitalizations and mortality rates from stroke increase exponentially with advancing age, studies on stroke in the pediatric population in Brazil are less common than in adults. Therefore, new studies are essential to monitor trends in risk factors, quality of health services, and socioeconomic conditions in Brazil, which may change this scenario in the long term.

During this period, the trend in childhood stroke mortality decreased in Brazil. However, the behavior of this reduction varies heterogeneously according to the federative units. Studies on the influence of socioeconomic factors on temporal stroke trends should be considered for further analyses. In addition, more epidemiological research with global and regional initiatives is required to improve pediatric health planning.

## Supplementary Information

Supplementary Tables.

## Data Availability

All data generated or analysed during this study are included in this published article.

## References

[1] Ferriero DM (2019). Management of stroke in neonates and children: A scientific statement from the American Heart Association/American Stroke Association. Stroke.

[2] Krishnamurthi RV (2015). Stroke prevalence, mortality and disability-adjusted life years in children and youth aged 0–19 years: Data from the Global and Regional Burden of Stroke 2013. Neuroepidemiology.

[3] GBD Stroke Collaborators (2019). Global, regional, and national burden of stroke and its risk factors, 1990–2019: A systematic analysis for the Global Burden of Disease Study 2019. Lancet Neurol..

[4] Catenaccio E (2020). Performance of a pediatric stroke alert team within a comprehensive stroke center. J. Child. Neurol..

[5] Ladner TR (2015). Pediatric acute stroke protocol activation in a children’s hospital emergency department. Stroke.

[6] Mastrangelo M (2022). Acute ischemic stroke in childhood: A comprehensive review. Eur. J. Pediatr..

[7] Virani SS (2020). Heart disease and stroke statistics-2020 update: A report from the American Heart Association. Circulation.

[8] Felling RJ, Sun LR, Maxwell EC, Goldenberg N, Bernard T (2017). Pediatric arterial ischemic stroke: Epidemiology, risk factors, and management. Blood Cells Mol. Dis..

[9] Gardner MA, Hills NK, Sidney S, Johnston SC, Fullerton HJ (2010). The 5-year direct medical cost of neonatal and childhood stroke in a population-based cohort. Neurology.

[10] Kossorotoff M, Chabrier S, Tran Dong K, Nguyen The Tich S, Dinomais M (2020). Arterial ischemic stroke in non-neonate children: Diagnostic and therapeutic specificities. Rev. Neurol..

[11] Fullerton HJ, Chetkovich DM, Wu YW, Smith WS, Johnston SC (2002). Deaths from stroke in US children, 1979 to 1998. Neurology.

[12] Lanthier S, Carmant L, David M, Larbrisseau A, de Veber G (2000). Stroke in children: the coexistence of multiple risk factors predicts poor outcome. Neurology.

[13] Fox CK, Johnston SC, Sidney S, Fullerton HJ (2012). High critical care usage due to pediatric stroke: Results of a population-based study. Neurology.

[14] DeVeber GA (2017). Epidemiology and outcomes of arterial ischemic stroke in children: The Canadian Pediatric Ischemic Stroke Registry. Pediatr. Neurol..

[15] Kirton A, deVeber G (2015). Paediatric stroke: Pressing issues and promising directions. Lancet Neurol..

[16] Mello GAM, Bridi BPL, Oliveira DC, Jantsch LB (2020). Prevalence of hospitalizations for stroke in children and adolescents. RSD..

[17] Ministério da Saúde do Brasil. Secretaria de Atenção à Saúde. *Departamento de Ações Programáticas Estratégicas. Diretrizes de Atenção à Reabilitação da Pessoa com Acidente Vascular Cerebral*. https://bvsms.saude.gov.br/bvs/publicacoes/diretrizes_atencao_reabilitacao_acidente_vascular_cerebral.pdf (2013).

[18] Martins SCO (2021). Fighting against stroke in Latin America: A joint effort of medical professional societies and governments. Front. Neurol..

[19] GBD 2019 Diseases and Injuries Collaborators (2020). Global burden of 369 diseases and injuries in 204 countries and territories, 1990–2019: A systematic analysis for the Global Burden of Disease Study 2019. Lancet.

[20] Global Health Data Exchange. GBD 2019 https://ghdx.healthdata.org/ (2022).

[21] Adami F (2016). Mortality and incidence of hospital admissions for stroke among Brazilians aged 15 to 49 years between 2008 and 2012. PLoS ONE.

[22] Aarnio K (2018). Return to work after ischemic stroke in young adults: A registry-based follow-up study. Neurology.

[23] Antunes JLF, Cardoso MRA (2015). Using time series analysis in epidemiological studies. Epidemiol. Serv. Saúde..

[24] Nugem R (2020). Stroke care in Brazil and France: National policies and healthcare indicators comparison. J. Multidiscip. Healthc..

[25] Rasella D, Harhay MO, Pamponet ML, Aquino R, Barreto ML (2014). Impact of primary health care on mortality from heart and cerebrovascular diseases in Brazil: A nationwide analysis of longitudinal data. BMJ.

[26] Bastos ML, Menzies D, Hone T, Dehghani K, Trajman A (2017). The impact of the Brazilian family health strategy on selected primary care sensitive conditions: A systematic review. PLoS ONE.

[27] Sporns PB (2022). Childhood stroke. Nat. Rev. Dis. Primers.

[28] IBGE – Instituto Brasileiro de Geografia e Estatística. *Censo Brasileiro de 2010*. (IBGE, 2012).

[29] Vincens N, Stafström M (2015). Income inequality, economic growth and stroke mortality in Brazil: Longitudinal and regional analysis 2002–2009. PLoS ONE.

[30] GBD 2019 Brazil Collaborators (2018). Burden of disease in Brazil, 1990–2016: A systematic subnational analysis for the Global Burden of Disease Study. Lancet.

[31] Oliveira GMM (2020). Estatística Cardiovascular – Brasil 2020. Arq Bras Cardiol..

[32] Ichihara GMM (2022). Mortality inequalities measured by socioeconomic indicators in Brazil: a scoping review. Rev. Saude Publ..

[33] Han CH, Kim H, Lee S, Chung JH (2019). Knowledge and poor understanding factors of stroke and heart attack symptoms. Int. J. Environ. Res. Public Health..

[34] Simmons C, Noble JM, Leighton-Herrmann E, Hecht MF, Williams O (2017). Community level measures of stroke knowledge among children: Findings from hip hop stroke. J. Stroke Cerebrovasc. Dis..

[35] Calderaro M (2022). The lack of knowledge on acute stroke in Brazil: A cross-sectional study with children, adolescents, and adults from public schools. Clinics.

[36] Barry M, Le TM, Gindville MC, Jordan LC (2018). In-hospital pediatric stroke alert activation. Pediatr Neurol..

[37] Andersen KK, Olsen TS (2019). Social inequality by income in short- and long-term cause-specific mortality after stroke. J. Stroke Cerebrovasc. Dis..

[38] Jordan LC (2018). Socioeconomic determinants of outcome after childhood arterial ischemic stroke. Neurology.

[39] França E (2014). Ill-defined causes of death in Brazil: A redistribution method based on the investigation of such causes. Rev. Saude Publ..

