# Supplementary material for: Changes in childhood stroke mortality from 1990 to 2019 in Brazil and its federative units
Source: Sci Rep. 2022 Dec 1;12:20757. doi: 10.1038/s41598-022-24761-x (PMC9715677; doi:10.1038/s41598-022-24761-x)
Supplement: Supplementary file 1 — Supplementary Tables. [file 41598_2022_24761_MOESM1_ESM.docx]

**Supplementary Tables S1, S2 and S3.**

**Table S1.** Temporal variation of ischemic stroke mortality rate (IS) according to localities, from 1990—2019­.

| **Ischemic Stroke (IS)** | **APC (CI 95%)** | **r²** | ***p*** | **Trend** |
| --- | --- | --- | --- | --- |
| **Federative Units** |  |  |  |  |
| Acre | -5.6 (-6.1; -5.0) | 0.93 | <0.001 | D |
| Alagoas | -8.5 (-9.9; -7.0) | 0.15 | <0.001 | D |
| Amapá | -2.3 (-4.6; 0.1) | 0.73 | 0.056 | S |
| Amazonas | -4.0 (-5.7; -2.3) | 0.74 | <0.001 | D |
| Bahia | -5.9 (-7.0; -4.8) | 0.76 | <0.001 | D |
| Ceará | -7.5 (-9.0; -6.1) | 0.37 | <0.001 | D |
| Distrito Federal | -5.0 (-6.4; -3.6) | 0.88 | <0.001 | D |
| Espírito Santo | -4.7 (-6.2; -3.2) | 0.77 | <0.001 | D |
| Goiás | -5.1 (-7.2; -3.1) | 0.56 | <0.001 | D |
| Maranhão | -9.2 (-10.3; -8.1) | 0.53 | <0.001 | D |
| Mato Grosso | -5.0 (-6.0; -3.9) | 0.89 | <0.001 | D |
| Mato Grosso do Sul | -4.9 (-5.8; -3.9) | 0.93 | <0.001 | D |
| Minas Gerais | -5.1 (-5.8; -4.4) | 0.95 | <0.001 | D |
| Pará | -4.1 (-5.2; -3.0) | 0.85 | <0.001 | D |
| Paraíba | -6.9 (-7.9; -6.0) | 0.8 | <0.001 | D |
| Paraná | -4.2 (-5.4; -3.1) | 0.89 | <0.001 | D |
| Pernambuco | -6.6 (-7.9; -5.4) | 0.65 | <0.001 | D |
| Piauí | -6.2 (-7.5; -5.0) | 0.57 | <0.001 | D |
| Rio de Janeiro | -5.5 (-7.3; -3.7) | 0.64 | <0.001 | D |
| Rio Grande do Norte | -6.7 (-7.8; -5.5) | 0.72 | <0.001 | D |
| Rio Grande do Sul | -3.8 (-5.1; -2.5) | 0.86 | <0.001 | D |
| Rondônia | -3.3 (-4.7; -1.9) | 0.83 | <0.001 | D |
| Roraima | -2.2 (-2.8; -1.6) | 0.87 | <0.001 | D |
| São Paulo | -4.3 (-5.8; -2.8) | 0.83 | <0.001 | D |
| Santa Catarina | -4.7 (-5.8; -3.6) | 0.89 | <0.001 | D |
| Sergipe | -7.1 (-8.5; -5.8) | 0.48 | <0.001 | D |
| Tocantins | -6.3 (-7.4; -5.2) | 0.8 | <0.001 | D |

APC, annual percentage change; r², predictive capacity of the model; *p,* probability; D, decreasing trend; S, stationary trend.

**Table S2.** Temporal variation of the mortality rate from intracerebral hemorrhage (IH) according to localities, from 1990—2019­.

| **Intracerebral Hemorrhage (IH)** | **APC (CI 95%)** | **r²** | ***p*** | **Trend** | |
| --- | --- | --- | --- | --- | --- |
| **Federative Units** |  |  |  |  |  |
| Acre | -4.7 (-5.4; -4.0) | 0.81 | <0.001 | D |  |
| Alagoas | -8.0 (-9.3; -6.6) | 0.6 | <0.001 | D |  |
| Amapá | -1.6 (-3.5; 0.3) | 0.52 | 0.101 | S |  |
| Amazonas | -2.6 (-3.5; -1.6) | 0.63 | <0.001 | D |  |
| Bahia | -4.8 (-6.2; -3.3) | 0.17 | <0.001 | D |  |
| Ceará | -7.7 (-9.1; -6.3) | 0.47 | <0.001 | D |  |
| Distrito Federal | -4.4 (-5.2; -3.6) | 0.85 | <0.001 | D |  |
| Espírito Santo | -3.6 (-5.0; -2.2) | 0.46 | 0.001 | D |  |
| Goiás | -4.3 (-5.8; -2.8) | 0.37 | <0.001 | D |  |
| Maranhão | -8.3 (-9.6; -6.9) | 0.66 | <0.001 | D |  |
| Mato Grosso | -4.1 (-4.9; -3.2) | 0.8 | <0.001 | D |  |
| Mato Grosso do Sul | -4.0 (-5.0; -3.0) | 0.76 | <0.001 | D |  |
| Minas Gerais | -4.0 (-5.2; -2.8) | 0.54 | <0.001 | D |  |
| Pará | -2.8 (-4.5; -1.1) | 0.26 | 0.003 | D |  |
| Paraíba | -6.5 (-7.1; -6.0) | 0.92 | <0.001 | D |  |
| Paraná | -3.0 (-4.3; -1.7) | 0.7 | <0.001 | D |  |
| Pernambuco | -5.5 (-7.2; -3.7) | 0.2 | <0.001 | D |  |
| Piauí | -6.0 (-7.3; -4.8) | 0.3 | <0.001 | D |  |
| Rio de Janeiro | -4.4 (-5.5; -3.3) | 0.46 | <0.001 | D |  |
| Rio Grande do Norte | -5.8 (-6.6; -5.0) | 0.63 | <0.001 | D |  |
| Rio Grande do Sul | -3.1 (-3.5; -2.7) | 0.9 | <0.001 | D |  |
| Rondônia | -2.2 (-3.4; -1.0) | 0.64 | 0.001 | D |  |
| Roraima | -1.2 (-1.9; -0.5) | 0.7 | 0.002 | D |  |
| São Paulo | -3.5 (-4.3; -2.8) | 0.8 | <0.001 | D |  |
| Santa Catarina | -3.8 (-4.6; -2.9) | 0.77 | <0.001 | D |  |
| Sergipe | -6.2 (-7.1; -5.2) | 0.61 | <0.001 | D |  |
| Tocantins | -6.1 (-7.3; -4.8) | 0.42 | <0.001 | D |  |

APC, annual percentage change; r², predictive capacity of the model; *p*, probability; D, decreasing trend; S, stationary trend.

**Table S3.** Mortality temporal variation from subarachnoid hemorrhage (SAH) according to localities, from 1990—2019­.

| **Subarachnoid Hemorrhage (SAH)** | **APC (CI 95%)** | **r²** | ***p*** | **Trend** |
| --- | --- | --- | --- | --- |
| **Federative Units** |  |  |  |  |
| Acre | -5.8 (-6.3; -5.2) | 0.65 | <0.001 | D |
| Alagoas | -6.4 (-7.5; -5.2) | 0.49 | <0.001 | D |
| Amapá | -0.8 (-2.5; 0.9) | 0.6 | 0.857 | S |
| Amazonas | -1.6 (-2.6; -0.7) | 0.62 | 0.095 | S |
| Bahia | -3.8 (-4.9; -2.6) | 0.17 | <0.001 | D |
| Ceará | -6.0 (-7.2; -4.8) | 0.26 | <0.001 | D |
| Distrito Federal | -3.3 (-4.0; -2.7) | 0.88 | <0.001 | D |
| Espírito Santo | -2.7 (-3.8; -1.5) | 0.47 | 0.001 | D |
| Goiás | -3.4 (-4.7; -2.1) | 0.42 | <0.001 | D |
| Maranhão | -6.5 (-7.6; -5.4) | 0.47 | <0.001 | D |
| Mato Grosso | -2.9 (-3.6; -2.2) | 0.74 | <0.001 | D |
| Mato Grosso do Sul | -3.0 (-3.7; -2.2) | 0.76 | <0.001 | D |
| Minas Gerais | -3.1 (-4.0; -2.2) | 0.5 | <0.001 | D |
| Pará | -1.8 (-3.2; -0.3) | 0.28 | 0.315 | S |
| Paraíba | -5.0 (-5.4; -4.5) | 0.93 | <0.001 | D |
| Paraná | -2.1 (-3.1; -1.1) | 0.71 | 0.006 | D |
| Pernambuco | -4.4 (-5.8; -2.9) | 0.17 | <0.001 | D |
| Piauí | -4.6 (-5.6; -3.5) | 0.22 | <0.001 | D |
| Rio de Janeiro | -3.3 (-4.3; -2.3) | 0.39 | <0.001 | D |
| Rio Grande do Norte | -4.6 (-5.1; -4.0) | 0.65 | <0.001 | D |
| Rio Grande do Sul | -2.3 (-2.7; -1.8) | 0.91 | <0.001 | D |
| Rondônia | -1.2 (-2.3; -0.1) | 0.7 | 0.544 | S |
| Roraima | -0.4 (-0.9; 0.2) | 0.65 | 0.170 | S |
| São Paulo | -2.6 (-3.2; -1.9) | 0.78 | <0.001 | D |
| Santa Catarina | -2.8 (-3.5; -2.0) | 0.78 | <0.001 | D |
| Sergipe | -4.9 (-5.7; -4.1) | 0.56 | <0.001 | D |
| Tocantins | -4.6 (-5.7; -3.6) | 0.24 | <0.001 | D |

APC, annual percentage change; r², predictive capacity of the model; *p*, probability; D, decreasing trend; S, stationary trend.
